# Directional beaming of light from a subwavelength metal slit with phase-gradient metasurfaces

**DOI:** 10.1038/s41598-017-09726-9

**Published:** 2017-09-21

**Authors:** Hua Zhu, Xiang Yin, Lin Chen, Xun Li

**Affiliations:** Wuhan National Laboratory for Optoelectronics, Huazhong University of Science and Technology, Wuhan, 430074 China

## Abstract

In this article, we demonstrate directional beaming of light from a metal nanoslit surrounded with phase-gradient metasurfaces on both sides. Distinct from the grating-based beaming structures, here the momentum mismatch between the surface wave and radiation wave is overcome by the phase-gradient metasurfaces. The deviation angle of the directional beam can be flexibly adjusted by appropriately arranging the phase-gradient of metasurfaces on each side of the nanoslit. The metasurface-based beaming structures also present the ability to operate with high diffraction efficiency and small divergence angle, implying various potential applications in nanophotonics.

## Introduction

Efficiently controlling the flow and propagation of light has always been the main theme in the field of optics. According to the classical diffraction theory, light passing through a subwavelength slit will diffract in all directions uniformly^1^. However, it is highly desirable for many applications to redirect light in a desired direction as a collimated beam, which has the great potential to promote the development of optical sensors, high-density optical data storage, divergence-controlled lasers, and so forth. Nevertheless, the surface wave (SW) on a metal surface cannot couple directly into the air due to the momentum mismatch between the radiation wave and SW. The utilization of gratings on a metal surface offers an approach to address this issue by providing an additional momentum so that the momentum mismatch is compensated. Further arrangement of the gratings surrounding a subwavelength metallic aperture allows for the realization of directional beaming of light^2–10^. Since the pioneering work on directional beaming by H. J. Lezec *et al*.^2^, much effort has been devoted to improving the beaming performance by optimizing the profile of the gratings^3–10^.

In recent years, the development of metasurface, an optically thin layer consisting of a monolayer of metal or dielectric antenna, has provided an unprecedented way to locally manipulate the phase and amplitude of the scattered electromagnetic field. The optical response of the metasurfaces can be designed to exhibit desired amplitude and phase by changing antennas geometry. Metasurfaces have enabled a variety of unique phenomena and applications that are unattainable with conventional materials, including anomalous reflection/refraction^11–17^, planar optical lenses^15, 18–20^, polarization converters^21–23^, vortex plates^24, 25^, ultrathin high-resolution holograms^26, 27^, and enhancement of nonlinear optical responses^28, 29^. Especially, recent study has demonstrated that, a gradient-index metasurface has provided an alternative approach to compensate the momentum mismatch between the radiation wave and SW, and hence high-efficiency coupling between them is achievable^16, 30^. According to the principle of optical path reversibility, the SW can also be converted to the radiation wave by properly arranging the gradient-index metasurfaces^31^. However, to the best of our knowledge, so far the potential for the utilization of metasurfaces to generate directional beaming of light from a subwavelength metal nanoslit has not been reported yet. In this article, we demonstrate that well-designed phase-gradient metasurfaces surrounding a metal nanoslit can be used to realize directional beaming of light. Distinct from the grating-based beaming structures, here the momentum mismatch between the SW and radiation wave is compensated by the phase-gradient metasurfaces. The deviation angle of the directional beam can be flexibly tuned by appropriately designing the phase-gradient of metasurfaces. We further demonstrate that, such kinds of beaming structures provide the benefits of high diffraction efficiency and small divergence angle.

## Results and Discussion

### Design of the phase-gradient metasurfaces

**Figure 1:**
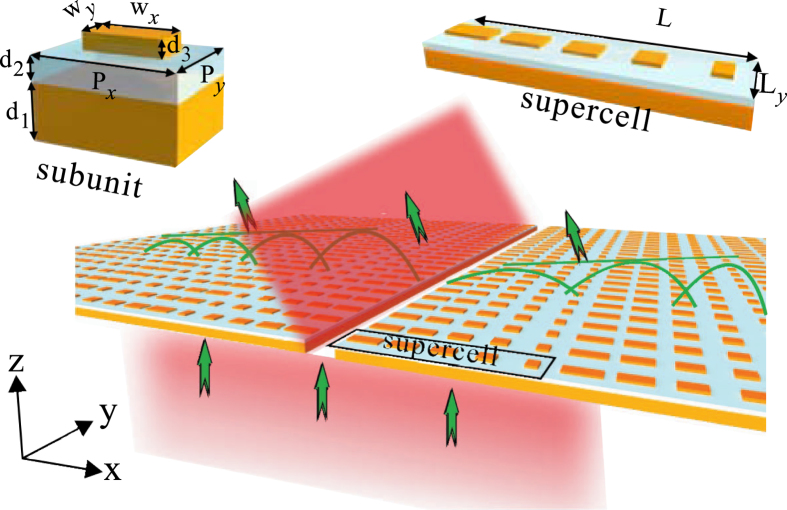
Schematic of the beaming structure, where a metal nanoslit is surrounded by phase-gradient metasurfaces on both sides. The number of the supercell on each side is *N*. A supercell of the metasurfaces (upper-right corner) contains several subunits (upper-left corner), each of which is comprised of a metal nanorod (yellow) and a metal film separated by a dielectric spacer (light blue). For the supercell, the lattice constant along x, and y direction is denoted as *L*_*x*_ and *L*_*y*_, respectively. The period of the subunit along the x and y direction is represented by *P*_*x*_ and *P*_*y*_, respectively. The thickness of the metal sheet, dielectric spacer, and metal nanorods is denoted as *d*_1_, *d*_2_ and *d*_3_, respectively. The width of the nanorod along x and y direction is represented by *w*_*x*_ and *w*_*y*_, respectively.

Figure 1 schematically shows the proposed beaming structure, where a subwavelength metal slit is surrounded by phase-gradient metasurfaces on both sides. For a subunit structure [upper-left corner in Fig. 1], electric current will be induced on both the metal nanorod and the ground metal plane when it is illuminated by an incident light polarized along the nanorod. Since the thickness of the dielectric spacer is much smaller than light wavelength, strong near-field coupling occurs, and thus strong magnetic field is generated inside the dielectric spacer^12^. The reflection phase of the subunit structure is highly dependent on the geometrical parameters, especially the width of the nanorod along the x direction. For a supercell [upper-right corner in Fig. 1], it consists of several subunit structures with the width of nanorod along the x direction gradually varied. On one hand, such a structure made of periodic array of metallic nanorods on a continuous metallic slab will provide a transverse wave-vector to the incoming light wave, when each subunit radiates with a different phase, *φ*, and the phase-gradient, *dφ/dx*, is constant within a supercell. On the other hand, similar to plasmonic mode on a continuous metallic slab, the surface plasmon-like mode can be supported and its dispersion relation can be significantly modified with the involvement of periodical metallic nanorods^32–35^. This surface plasmon-like mode, also termed as SW by several groups, has been widely used for diverse interesting applications such as Luneburg Lens^33^, planar electromagnetic devices/circuits^34^, and “transform optics”^35^.

According to the generalized Snell’s law, the transverse wave vectors at the two interfaces must satisfy the relation as^11^

1 $${k}_{0}\,\sin \,{\theta }_{r}-{k}_{i}\,\sin \,{\theta }_{i}=d\phi /dx$$

where *k*_0_ is the propagation constant of radiation light in the air, *k*_*i*_ is the wave vector of the incident light, and *θ*_*r*_ (*θ*_*i*_) denotes the radiation (incident) angle. Assuming a TM polarized light (electric filed polarized along x-direction) is incident from the bottom of the nanoslit of the beaming structure shown in Fig. 1, it will be diffracted from the nanoslit, and then converted to the SW propagating along the metasurfaces with the propagation constant, *k*_*sw*_. In this case, the SW contributes a momentum of *k*_*sw*_ in the transverse direction, and thus the second term, ${k}_{i}\,\sin \,{\theta }_{i}$, in Eq. (1) should be taken place by *k*_*sw*_. As a result, the generalized Snell’s law for the current case can be rewritten as

2 $${k}_{0}\,\sin \,\theta ={k}_{sw}+d\phi /dx$$

Here, *θ* denotes the radiation angle. It is highly anticipated that the SW can be converted to the radiation wave redirected at a specific radiation angle, *θ*.

**Figure 2:**
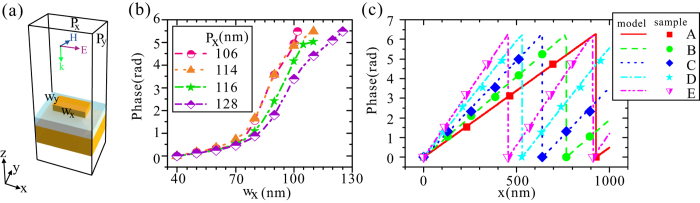
(**a**) A unit cell of the uniform metasurface. (**b**) The dependence of the reflection-phase of the metasurface on *w*_*x*_ for different *P*_*x*_. (**c**) The reflection phase of each subunit structure within a supercell for five different supercells. For the subunit structure, *d*_1_, *d*_2_, *d*_3_, *L*_*y*_, *P*_*y*_ and *w*_*y*_ are fixed at 200, 60, 30, 240, 240, and 35 nm, respectively, while the other structural parameters are given in Table 1. Ag and MgF_2_ are selected as the metal layer and dielectric spacer with the relative permittivities of −27.92 + 1.52i and 1.9 at 800 nm^37^, respectively.

**Figure 3:**
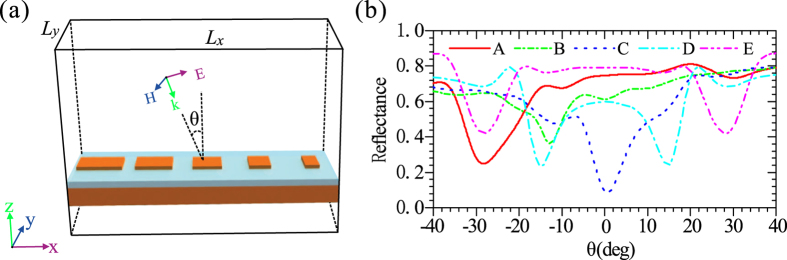
(**a**) Schematic of a supercell of the phase gradient metasurface. (**b**) The dependence of the reflectance of the metasurfaces A-E on the incident angle when a TM polarized plane wave illuminates the metasurface.

**Figure 4:**
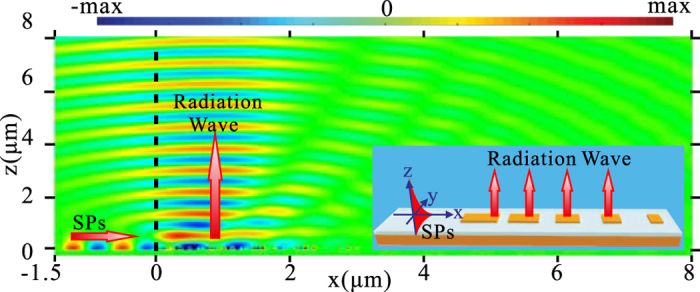
FDTD simulated Ex field pattern on the x-z plane when SPs is excited on the left-hand side and propagate towards the region of metasurfaces C on the right-hand side. The black dashed line represents the interface of the SP waveguide and metasurface region. 20 supercells are used in the simulation. The inset schematically shows the configuration of the investigated structure.

In order to design the desired phase gradient metasurfaces, we firstly calculate the reflection phase as a function of the width of the nanorod, w_x_, for a uniform metasurfaces structure [see its unit cell in Fig. 2(a)], which is illuminated by a plane wave from the top. It can be clearly seen from Fig. 2(b) that the reflection phase of a uniform metasurface structure can be adjusted to be an arbitrary value in the range of 0-2π by varying *w*_*x*_. Further, different phase-gradient, $d\phi /dx$, within a supercell can be made by properly choosing different subunit structures to form a supercell [Fig. 2(c)]. The phase-gradients for the five supercells A, B, C, D and E in Fig. 2(c) are 0.86 *k*_0_, $1.04\,{k}_{0}$, $1.25\,{k}_{0}$, $1.51\,{k}_{0}$, and $1.75\,{k}_{0}$, respectively. Due to the different structural parameters used, the propagation constant, ${k}_{sw}$, is distinct for different metasurfaces, of which a supercell is schematically shown in Fig. 3(a). In order to retrieve the propagation constant, *k*_*sw*_ finite difference time domain (FDTD) simulation has been performed to calculate the dependence of the reflectance characteristics on the incident angle [Fig. 3]. In the simulations, a supercell shown in Fig. 3(a) is used and the reflectance represents the ratio of the power of the reflected light and the power of the incident light. When the incident light provides a transverse wave vector (${k}_{0}\,\sin \,\theta$) so that Eq. (2) is satisfied, a portion of light energy will be converted into the SW mode, resulting in the presence of a notable dip in the reflectance curve. Consequently, the propagation constant of the SW mode, ${k}_{sw}$, can be retrieved by incorporating the corresponding $\theta$, associated with the reflection dip, into Eq. (2). It can be observed that different metasurfaces have different reflection dips, associated with the incident angles. The propagation constants of the SW, ${k}_{sw}$, for five metasurfaces (A–E) are $1.33\,{k}_{0}$, $1.27\,{k}_{0}$, 1.25 *k*_0_, $1.28\,{k}_{0}$, and $1.28{k}_{0}$, respectively. It should be emphasized here, lower reflection [Fig. 3(b)] means that more energy of incident light is coupled into the SW. Consequently, it can be inferred that, by utilization of metasurface C the SW can be more efficiently converted into the radiation wave in view of the principle of optical path reversibility. We then consider the reversible process above-mentioned, i.e., the SW is converted to the radiation wave with a specific diffraction angle when the momentum mismatch between the SW and radiation wave is overcome by utilization of phase-gradient metasurfaces. Consider that surface plasmons (SPs) is launched on the left-hand side, and propagates towards the region of metasurfaces C [Fig. 4]. It firstly propagates along the plasmonic waveguide, and then is coupled to the SW mode in the metasurface region, and finally converted to the radiation wave with the direction normal to the metasurface. The radiation angle retrieved from the simulation data is well consistent with that predicted by Eq. (2).

**Table 1:** Structural parameters for the five supercells.

No	L_x_(nm)	P_x_ (nm)	w_x_ (nm) in each supercell
A	928	116	40, 40, 84, 84, 94, 94, 104, 104
B	768	128	40, 83, 92, 98, 107, 122
C	640	128	40, 85, 93, 102, 118
D	530	106	40, 78, 85, 90, 99
E	456	114	40, 79, 88, 99

### Phase-gradient metasurfaces for directional beaming

**Figure 5:**
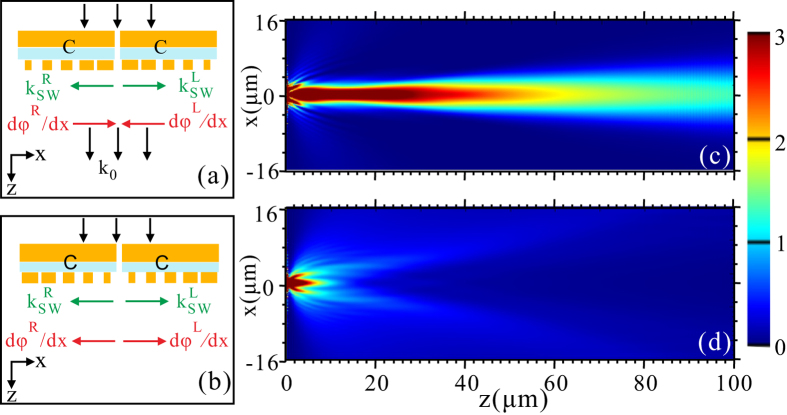
Field intensity distributions of |*E*|^2^ (**a**) and (**b**) Schematic of a metal nanoslit surrounded by metasurfaces C symmetrically placed on both sides. The phase-gradient, $d\phi /dx$, has the opposite (**a**) and same (**b**) direction to the SW. (**c**) and (**d**) Field intensity distributions of |*E*|^2^ when the two structures are illuminated with a plane wave from the bottom of the nanoslit (100 nm width). 20 supercells of the metasurfaces are used on each side of the nanoslit.

We next consider the utilization of metasurfaces to surround the metal nanoslit [Fig. 1] to enable the directional beaming of light. Metasurfaces C is symmetrically placed on both sides of the metal nanoslit, while the phase-gradient, $d\phi /dx$, has the opposite [Fig. 5(a)] and same [Fig. 5(b)] direction to the SW. The diffraction field for the two cases are presented in Fig. 5(c,d). It can be seen from Fig. 5(c) that, for the former case on-axis directional beam is realized. This can be explained as follows. The propagation constants for the left-going and right-going SW are ${k}_{sw}^{L}=-1.25\,{k}_{0}$ and ${k}_{sw}^{R}=-1.25\,{k}_{0}$, respectively. Meanwhile, the metasurfaces C on the left and right sides provides an additional transverse wave vector of $d{\phi }^{L}/dx=-1.25\,{k}_{0}$ and $d{\phi }^{R}/dx=1.25\,{k}_{0}$, respectively. As a result, the radiation angle is zero for both the left and right metasurfaces since we have ${k}_{sw}^{L}+d{\phi }^{L}/dx=0$, and ${k}_{sw}^{R}+d{\phi }^{R}/dx=0$. However, for the latter case no directional beaming effect is observed [Fig. 5(d)]. The wave vector offered by the left and right side metasurfaces is $d{\phi }^{L}/dx=1.25\,{k}_{0}$ and $d{\phi }^{R}/dx=-1.25\,{k}_{0}$, respectively. We thus have ${k}_{sw}^{L}+d{\phi }^{L}/dx=2.5\,{k}_{0}$ and ${k}_{sw}^{L}+d{\phi }^{L}/dx=-2.5\,{k}_{0}$. Consequently, the SW will be bounded on the metal surface and gradually diffracted without directivity. It should be noted that, the totally different results for the above-mentioned two cases indicate that the momentum mismatch is overcome by the phase-gradient of the metasurfaces, rather than the grating effects of the supercell of the metasurfaces.

**Figure 6:**
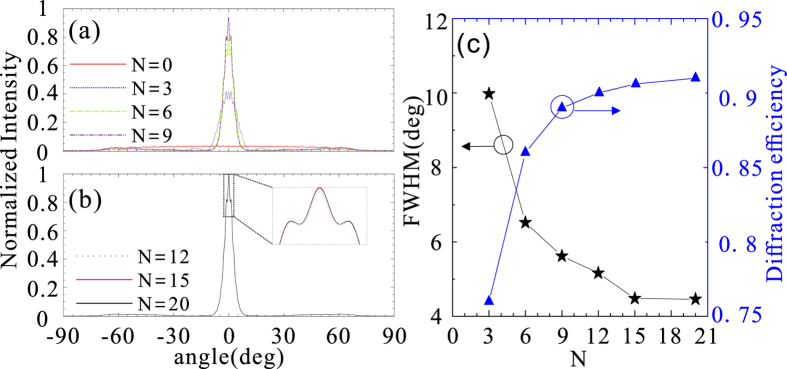
(**a**,**b**) The far-field profile of |*E*|^2^ versus the angle for the diffraction field with different number of the supercell, N, on each side of the nanoslit. (**c**) The FWHM of the far-field profiles of |*E*|^2^ and diffraction efficiency as a function of N.

Figure 6 shows how the number of the supercell of the metasurface, *N*, affects the far-field profile intensity, the divergence angle and diffraction efficiency for the case of on-axis beaming. Here, the divergence angle is defined as the full width at half maximum (FWHM) of the far-field angle distribution curve, and the diffraction efficiency, ${\eta }_{D}$, is defined as the percentage of incoming power distributed in the central lobe of angular spectrum confined by the angles of two closely adjacent minimum^7^

3 $${\eta }_{D}={I}_{d}/{I}_{out}$$

where ${I}_{d}$ and ${I}_{out}$ denote the power within the central lobe of the directional beam and the total transmitted power, respectively. It is apparent from Fig. 6(a) that the beam intensity is enhanced as more supercells contribute to the diffraction field. When the number of the supercell, *N*, is more than 15, the far-filed profile is almost unchanged due to the fact that the field intensity of the SW decays along the metal surface. In addition, the diffraction efficiency increases, and the divergence angle decreases as the number of the supercell is increased [Fig. 6(b)]. We have noted a previous study on the utilization of phase-gradient metasurfaces for converting the radiation wave into the SW^36^, where the conversion efficiency is reduced with the increase of supercell number, N. This is due to the fact that the abrupt inhomogeneity on the supercell boundaries introduces scattering loss for the SW, which thus significantly suppresses the conversion efficiency with the increased supercell number. By contrast, if more supercells are introduced to the beaming structure, the scattering wave arising from the supercell boundaries contributes to the power of the directional beam, which is beneficial for the far-field intensity and diffraction efficiency. The FWHM of the far-field is 4.4° for *N* = 20, which suggests a highly directional beam is achieved. The diffraction efficiency is found as high as 91% with *N* = 20, implying the intensity of side lobes is very weak with respect to the central beam [Fig. 5(c)].

**Figure 7:**
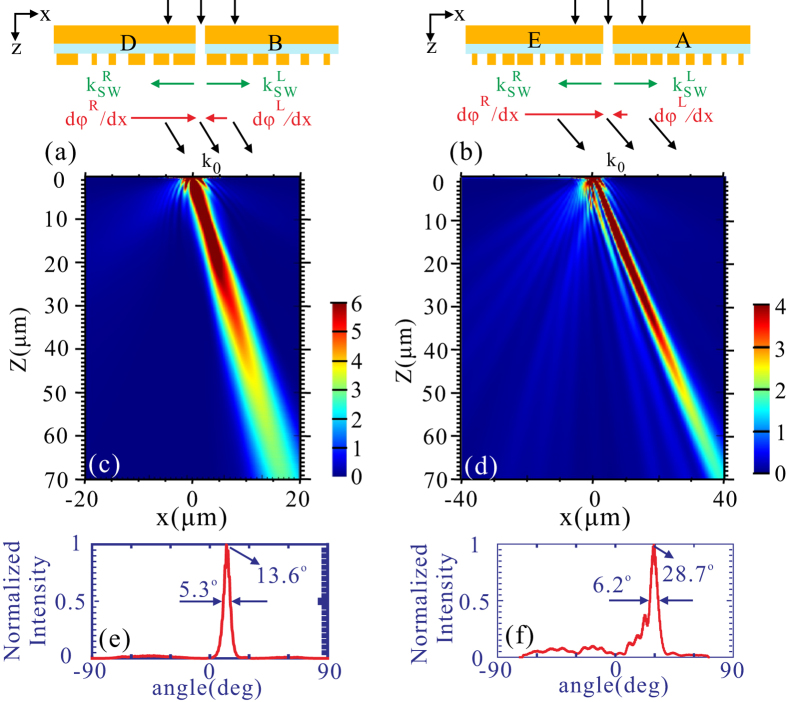
(**a**) and (**b**) Schematic of a metal nanoslit surrounded by different metasurfaces on both sides. (**a**) left-hand side: metasurface B, right-hand side: metasurface D, (**b**) left-hand side: metasurface A, right-hand side: metasurface E. (**c**) and (**d**) Field intensity distributions of |*E*|^2^ for (**a**) and (**b**). (**e**) and (**f**) Far-field profiles of |*E*|^2^ for (**c**) and (**d**), respectively. All the other structural parameters are the same as those in Fig. 5.

When different metasurfaces with different phase-gradients are asymmetrically arranged on both sides of the nanoslit, we are able to flexibly adjust the deviation angle of the diffraction field. If the metasurfaces on each side is tuned to have the same radiation angle (not zero), it is highly expected to achieve off-axis directional beaming. Fig. 7(a) and (b) schematically represent two sets of off-axis beaming structures, where the phase-gradient and the propagation constant of the SW for metasurfaces B, D, A, E are $d{\phi }^{L}/dx=-1.04\,{k}_{0}$, $d{\phi }^{R}/dx=1.51\,{k}_{0}$, $d{\phi }^{L}/dx=-0.86\,{k}_{0}$, $d{\phi }^{R}/dx=1.75\,{k}_{0}$ and ${k}_{sw}^{L}=1.27\,{k}_{0}$, ${k}_{sw}^{R}=-1.28\,{k}_{0}$, ${k}_{sw}^{L}=1.33\,{k}_{0}$, ${k}_{sw}^{R}=-1.28\,{k}_{0}$, respectively. Therefore, the total transverse wave vector of the diffraction field for the two structures are $0.23{k}_{0}$ and $0.47{k}_{0}$ respectively, associated with the deviation angle of 13.3° and 28.0°, respectively. The simulated field intensity shown in Fig. 7(c) and (d) reveals that the diffraction field is redirected into the air as a collimated beam, in which the deviation angles are well consistent with the prediction above-mentioned [Fig. 7(e) and (f)], and divergence angles are still kept at a low level (5.3° and 6.2°, respectively). It should be mentioned that the proportion of the diffraction field redirected into the undesired direction in Fig. 7(e) is more than that in Fig. 7(f), hence the diffraction efficiency is much smaller. To make the beaming structures operate with a much larger deviation angle, metasurface E with a higher phase gradient is used, but which is at the expense of lower diffraction efficiency^12^.

## Conclusion

In conclusion, we have demonstrated directional beaming of light from a metal nanoslit surrounded by phase-gradient metasurfaces. The momentum mismatch between the SW and radiation wave is overcome by the phase-gradient of the metasurfaces, rather than the grating effects. By properly designing the phase-gradient of the metasurfaces on each side of the nanoslit, we are able to flexibly adjust the deviation angle of the directional beam. In addition, the presented metasurface-based beaming structures offer the performance merits including high diffraction efficiency, and small divergence angle in the far-field, which may lead to a wide range of applications, including optical sensors, high-density optical data storage, and divergence-controlled lasers.

## Methods

In this work, the simulation results shown in Figs 2–7 were conducted by Lumerical finite difference time domain solutions. In Figs 2 and 3, Bloch boundary condition is employed in the x and y directions, and perfect matched layer absorption condition is applied in the z direction. In Figs 4, 5, 6 and 7, Bloch boundary condition is employed in the y direction, and perfect matched layer absorption condition is applied in the x and z directions.
